# Immune responses and transcription landscape of adults with the third dose of homologous and heterologous booster vaccines of COVID-19

**DOI:** 10.3389/fimmu.2024.1461419

**Published:** 2024-09-12

**Authors:** Hui Zheng, Cuidan Li, Xiuyu Zheng, Hu-Dachuan Jiang, Yuqing Li, Aihua Yao, Xiaolong Li, Feiyu Wang, Wenqing Liu, Xiang Cao, Runjie Qi, Li Chen, Lairun Jin, Fengcai Zhu, Jingxin Li, Fei Chen

**Affiliations:** ^1^ Key Laboratory of Environmental Medicine Engineering, Ministry of Education, School of Public Health, Southeast University, Nanjing, Jiangsu, China; ^2^ National Health Commission (NHC) Key Laboratory of Enteric Pathogenic Microbiology, Jiangsu Provincial Center for Disease Control and Prevention, Nanjing, Jiangsu, China; ^3^ Chinese Academy of Sciences (CAS) Key Laboratory of Genome Sciences and Information, Beijing Institute of Genomics, Chinese Academy of Sciences and China National Center for Bioinformation, Beijing, China; ^4^ Research and Development Department, CanSino Biologics Inc., Tianjin, China; ^5^ School of Public Health, Nanjing Medical University, Nanjing, Jiangsu, China

**Keywords:** Ad5-nCoV, COVID-19, aerosol inhalation, intramuscular injection, transcriptome, immunity

## Abstract

**Background:**

Heterologous booster vaccines are more effective than homologous booster vaccines in combating the coronavirus disease 2019 (COVID-19) outbreak. However, our understanding of homologous and heterologous booster vaccines for COVID-19 remains limited.

**Methods:**

We recruited 34 healthy participants from two cohorts who were primed with two-dose inactivated COVID-19 vaccine before, vaccinated with COVID-19 inactivated vaccine and adenovirus-vectored vaccine (intramuscular and aerosol inhalation of Ad5-nCoV) as a third booster dose. We assessed the immune responses of participants before and 14 days after vaccination, including levels of neutralizing antibodies, IgG, and cytokines, and quantified the transcriptional profile of peripheral blood mononuclear cells (PBMCs).

**Results:**

The Ad5-nCoV group showed a significantly higher neutralizing antibody geometric mean titer (GMT) compared to the ICV group after 14 days of heterologous boosting. The intramuscular Ad5-nCoV group had a GMT of 191.8 (95% CI 129.0, 285.1) compared to 38.1 (95% CI 23.1, 62.8) in the ICV_1_ group (p<0.0001). The aerosolized Ad5-nCoV group had a GMT of 738.4 (95% CI 250.9-2173.0) compared to 244.0 (95% CI 135.0, 441.2) in the ICV_2_ group (p=0.0434). Participants in the aerosolized Ad5-nCoV group had median IFN-γ+ spot counts of 36.5 (IQR 15.3-58.8) per 10^6^ PBMCs, whereas, both intramuscular Ad5-nCoV and CoronaVac immunization as the third dose showed lower responses. This suggests that a third dose of booster Ad5-nCoV vaccine (especially aerosolized inhalation) as a heterologous vaccine booster induces stronger humoral and cellular immune responses, which may be more potent against VOCs than the use of inactivated vaccine homologs. In transcriptomic analyses, both aerosolized inhalation/intramuscular injection of the Ad5-nCoV vaccine and inactivated vaccine induced a large number of differentially expressed genes that were significantly associated with several important innate immune pathways including inflammatory responses, regulation of the defense response, and regulation of cytokine production. In addition, we identified crucial molecular modules of protective immunity that are significantly correlated with vaccine type and neutralizing antibodies level.

**Conclusion:**

This study demonstrated that inhalation/intramuscular injection of the Ad5-nCoV vaccine-mediated stronger humoral and cellular immune responses compared with the inactivated vaccine, and correlated significantly with innate immune function modules, supporting a heterologous booster immunization strategy.

## Introduction

The COVID-19 pandemic, caused by the severe acute respiratory syndrome coronavirus 2 (SARS-CoV-2), has emerged as a significant global health and socioeconomic challenge. As of May 2, 2024, there have been over 775 million confirmed cases and more than 7.04 million deaths due to the disease worldwide (1). The administration of efficient COVID-19 vaccines is widely acknowledged as the most cost-effective strategy for preventing and managing the spread of COVID-19 (2, 3).

CanSino’s Ad5-nCoV vaccine is a replication-defective Ad5 vector vaccine that expresses the SARS-CoV-2 spike glycoprotein (S protein). It is administered either via intramuscular injection of 0.5 ml (5.0 × 10^10^ VP) into the deltoid muscle of the lateral upper arm or through aerosol inhalation of 0.1 ml (1.0 × 10^10^ VP) of the vaccine (4). Previous studies have demonstrated that both aerosol inhalation and intramuscular injection of the Ad5-nCoV vaccine, following the initial priming immunization with two or three doses of inactivated vaccines, resulted in significantly higher levels of neutralizing antibodies compared to those induced by the homologous inactivated vaccine (4–6). Specifically, the levels of neutralizing antibodies were found to be 5.9 times higher in individuals who received the Ad5-nCoV vaccine. Furthermore, aerosol inhalation of the Ad5-nCoV vaccine was shown to elicit robust Th1-type cellular immune responses and stimulate mucosal immunity (4). In a report detailing the emergence of COVID-19 variant Omicron breakthrough cases across various regions in China, the relative protection of the heterologous booster with aerosolized Ad5-nCoV versus homologous booster with inactivated COVID-19 vaccine was 35.1% (95% CI 23.0–45.2) about 12 months after the vaccination, which provides solid evidence that the superiority of protection associated with heterologous boosting strategy (7). The enhanced immunological protection conferred by heterologous booster immunization with the Ad5-nCoV vaccine remains a subject of limited understanding. The intricate mechanisms underlying the humoral and cellular immune responses elicited by Ad5-nCoV vaccination have yet to be fully elucidated. Further research is warranted to unravel the complexities of these immune responses and shed light on the reasons behind the superior efficacy observed with the Ad5-nCoV vaccine as a booster.

The utilization of high-throughput sequencing technologies and bioinformatics methodologies in systems biology analyses offers a novel tool for evaluating enhanced immune response following a heterologous booster immunization (8, 9). Through these advanced techniques, we enable the exploration of immune response dynamics and the identification of intricate gene regulatory networks, providing valuable insights into the mechanisms underlying vaccine-mediated immunity (10). Leveraging transcriptomics to investigate vaccines like Hantavirus, Influenza, VSV-EBOV, and BNT162b mRNA has facilitated a thorough analysis of the intricate immune responses elicited by vaccination in participants. This approach has enabled us to unravel the dynamics of complex immune reactions induced by these vaccines, showcasing the significant value of cutting-edge technologies in advancing vaccine research (11–14).

In this study, we present findings on the heterologous or homologous boosting immune responses and transcriptional landscape in immunized participants who received a third dose of either aerosolized inhalation or intramuscular injection of the Ad5-nCoV vaccine, as well as those who received the inactivated COVID-19 vaccine.

## Materials and methods

### Study design and participants

In this analysis, thirty-four participants from two randomized, parallel-controlled Phase IV clinical trials (NCT04892459; NCT05043259) were included. The study design, previously reported, involved 24 participants from cohort 1 (NCT04892459) who had been primed with two doses of inactivated COVID-19 vaccines and received either the Ad5-nCoV vaccine (IM-Ad5-nCoV group, n=12) or the CoronaVac inactivated vaccine (ICV_1_ group, n=12) via intramuscular injection. Additionally, 10 participants from cohort 2 (NCT05043259) who had been immunized with two-dose COVID-19 vaccines before, were vaccinated with the Ad5-nCoV vaccine through aerosol inhalation (IH-Ad5-nCov group, n=5) or the inactivated CoronaVac vaccine via intramuscular injection (ICV_2_ group, n=5). Blood samples were collected from the participants for immunogenicity assessment on day 0 (before booster vaccination) and day 14 post-booster vaccination (**Supplementary Figure 1**). The study protocol and informed consent procedures were reviewed and approved by the Research Ethics Committee of the Jiangsu Provincial Centre for Disease Control and Prevention, with written informed consent obtained from each participant before enrollment.

### Quantification of neutralizing antibodies, IgG, and cytokines

Serum and peripheral blood mononuclear cells (PBMCs) were isolated from venous blood samples. Neutralizing antibodies against the live SARS-CoV-2 virus were assessed through a cytopathic effect micro-neutralization assay, utilizing the wild-type SARS-CoV-2 strain BetaCoV/Jiangsu/JS02/2020 (GISAID EPI_ISL_411952) (5). In the assay, serum dilutions ranging from 1:4 to 1:8192 were mixed with an equal volume of virus solution to achieve a 50% tissue culture infectious dose of 100 per well. The reported titer represents the reciprocal of the highest sample dilution that, when observed under an inverted microscope, protects at least 50% of the cells from cytopathy. A live SARS-CoV-2 virus-neutralizing antibody titer of ≥1:4 is considered seropositive. Receptor-binding domain (RBD)-specific IgG titers were determined via ELISA using a commercially available anti-SARS-CoV-2 RBD IgG ELISA kit (Vazyme Medical Technology, Nanjing, China) with a titer threshold of 1:10[16]. Fresh PBMCs were stimulated with overlapping spike glycoprotein peptide pools, and the cytokines secreted by T-helper type 1 cells (Th1: IFN-γ) and T-helper type 2 cells (Th2: IL-13) were quantified in every 106 PBMCs using enzyme-linked immunospot (ELISpot) analysis.

Statistical analyses were conducted using SPSS (version 25) or R (version 4.3.1). Categorical data were analyzed using the χ2 test or Fisher’s exact test, log-transformed antibody titers were analyzed using the T-test, and data that did not follow a normal distribution were analyzed using the Wilcoxon rank-sum test. The serum neutralizing antibody (NAb) geometric mean titers (GMTs) of the heterologous boost group were compared to those of the homologous boost group, calculated with two-sided 95% confidence intervals (CIs) based on the t-distribution of the log-transformed titers, and then back-transformed to the original scale. RBD-specific binding antibodies were assessed for increases of fourfold or more compared to baseline levels prior to the booster vaccination. Data below the detection limit were assigned a value of half the threshold, while values above the highest detection limit were set to that limit for calculation purposes.

### mRNA library construction and transcriptome sequencing

PBMCs were cryopreserved in a cell preservation medium containing 10% DMSO and stored in liquid nitrogen. Upon resuscitation, the PBMCs were utilized for transcriptome sequencing. Total RNA extraction from PBMCs was carried out using the phenol/chloroform method, and the purity, concentration, and integrity of the total RNA were assessed. Samples that met the required criteria underwent library construction and subsequent sequencing. Briefly, total RNA was utilized for library preparation, with poly(A) mRNA being purified using oligo(dT) 25 magnetic beads. Subsequently, the mRNA was fragmented into small pieces using divalent cations at high temperatures, followed by the synthesis of first and second-strand cDNAs. The purified double-stranded cDNAs underwent end repair, dA-tailing, and T-A ligation to add aptamers to the ends. Size selection of the aptamer-ligated DNA was performed using clean DNA beads. PCR amplification and validation were conducted for each sample. The libraries were then sequenced on the Illumina NovaSeq 6000 platform following the manufacturer’s instructions.

### Differentially expressed gene analysis

The transcriptome count data before and after vaccination was analyzed using the R package *DESeq2* to identify differentially expressed genes (DEGs) (15). DEGs were determined based on a cut-off criterion of |log2FC|≥1.0 and an adjusted p-value of <0.05, with the fold change (FC) in gene expression converted to a logarithmic value. P-values were adjusted using the Benjamini-Hochberg method to control for false discovery rate (FDR). The DEGs were visualized as volcano plots using the R package *ggplot2*. Additionally, variance stabilized normalization (VSN) was employed to process the sequencing data for principal component analysis (PCA) for downscaling and quality control.

### Weighted gene co-expression network analysis explores gene modules associated with vaccine responses

In our analysis, we utilized the *WGCNA* package in R to transform gene expression data into gene co-expression networks for investigating highly correlated gene modules (16). To address batch effects, the *ComBat* function from the *sva* package was applied. The *pickSoftThreshold* function was used to determine a soft threshold for the transcriptome data. Subsequently, the neighbor-joining matrix was computed using the formula: a_ij_ = |S_ij_|^β^ (where a_ij_ represents the neighbor-joining matrix between gene I and gene J, S_ij_ denotes the similarity matrix calculated via Pearson correlation of all gene pairs, and β signifies the soft threshold). The neighbor-joining matrix was then converted into a topological overlap matrix (TOM) and its corresponding difference matrix (1-TOM). Next, the hierarchical clustering dendrogram of the 1-TOM matrix was employed to classify related genes into distinct co-expression modules and to explore the associations of these modules with the traits under study. Modules exhibiting high correlation coefficients were prioritized for further analysis, as they potentially have close ties to the vaccination process.

### Enrichment analysis of differentially expressed genes and coexpression modules

Gene Ontology (GO) analyses of differentially expressed genes (DEGs) and co-expression modules, covering biological processes (BP), molecular functions (MF), and cellular components (CC, were carried out using the *clusterProfiler* package in R software (17). Gene set enrichment analysis (GSEA) was conducted to unveil potential functional mechanisms, utilizing the *c2.cp.v7.2.symbols.gtm* file. Threshold values of False Discovery Rate (FDR) < 0.2 and normalized p-value < 0.05 were set. Additionally, the online tool Metascape was utilized for further enrichment analyses and the construction of enrichment term networks.

## Results

### Demographic characteristics

**Figure 1:**
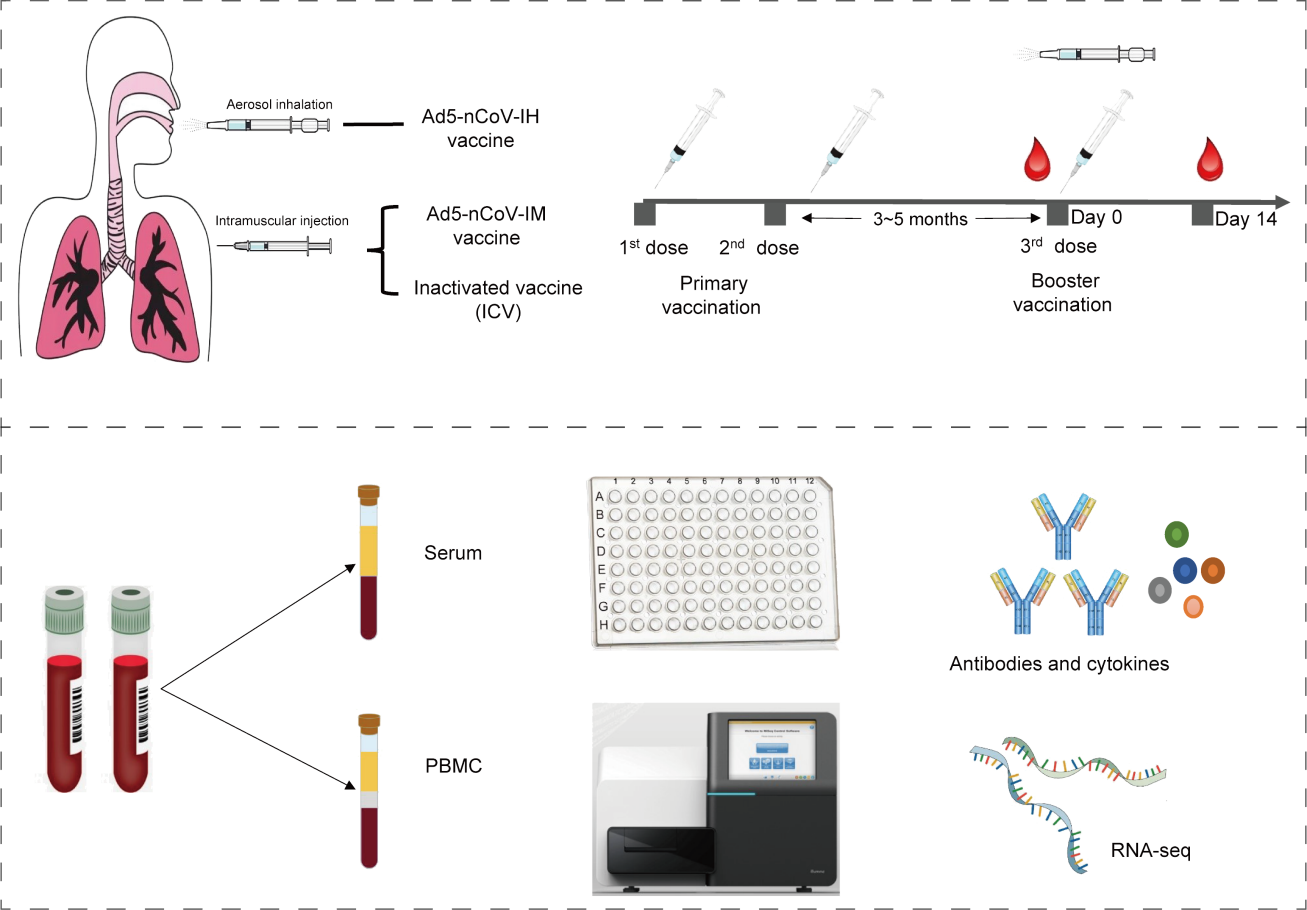
Study schematic.

The study workflow is depicted in **Figure 1**. A total of 34 participants who have been primed with two doses of inactivated COVID-19 vaccines were involved in this study, of which 5 received Ad5-nCoV by aerosol inhalation, 12 received Ad5-nCoV by intramuscular injection, and 17 (12 from cohort 1 and 5 from cohort 2) were vaccinated with the inactivated vaccine CoronaVac as a booster dose. The clinical characteristics of the participants are outlined in **Table 1**. The participants were all between 18 and 60 years old, with no history of COVID-19 infection, equally divided between males and females. In addition, the time intervals between the completion of the primary immunization with two doses of inactivated vaccine and the booster dose was 3.9 (IQR: 3.2, 4.6) months in cohort 1, compared with 5.0 (IQR: 5.0, 5.0) months in cohort 2.

**Table 1:** Baseline characteristics of the participants.

	Cohort 1: NCT04892459			Cohort 2: NCT05043259		
	IM-Ad5-nCoV	ICV_1_	P-value	IH-Ad5-nCoV	ICV_2_	P-value
**Number, n**	12	12		5	5	
**Age, Median age (IQR)**	46.5 (46.0, 48.0)	47.0 (46.0, 48.0)	0.8877	44.0 (29.0, 50.0)	43.0 (40.0, 47.0)	0.5818
Sex, %						
Female	6 (50.0)	6 (50.0)	>0.9999	3 (60.0)	3 (60.0)	>0.9999
Male	6 (50.0)	6 (50.0)		2 (40.0)	2 (40.0)	
**Time since the last priming dose of inactivated vaccine, months**	3.9 (3.2, 4.6)	3.9 (3.2, 4.6)	0.9933	5.0 (5.0, 5.0)	5.0 (5.0, 5.0)	>0.9999

Data are n (%), mean (SD), or median (IQR).

### Immune response to booster vaccination with the Ad5-nCoV

**Figure 2:**
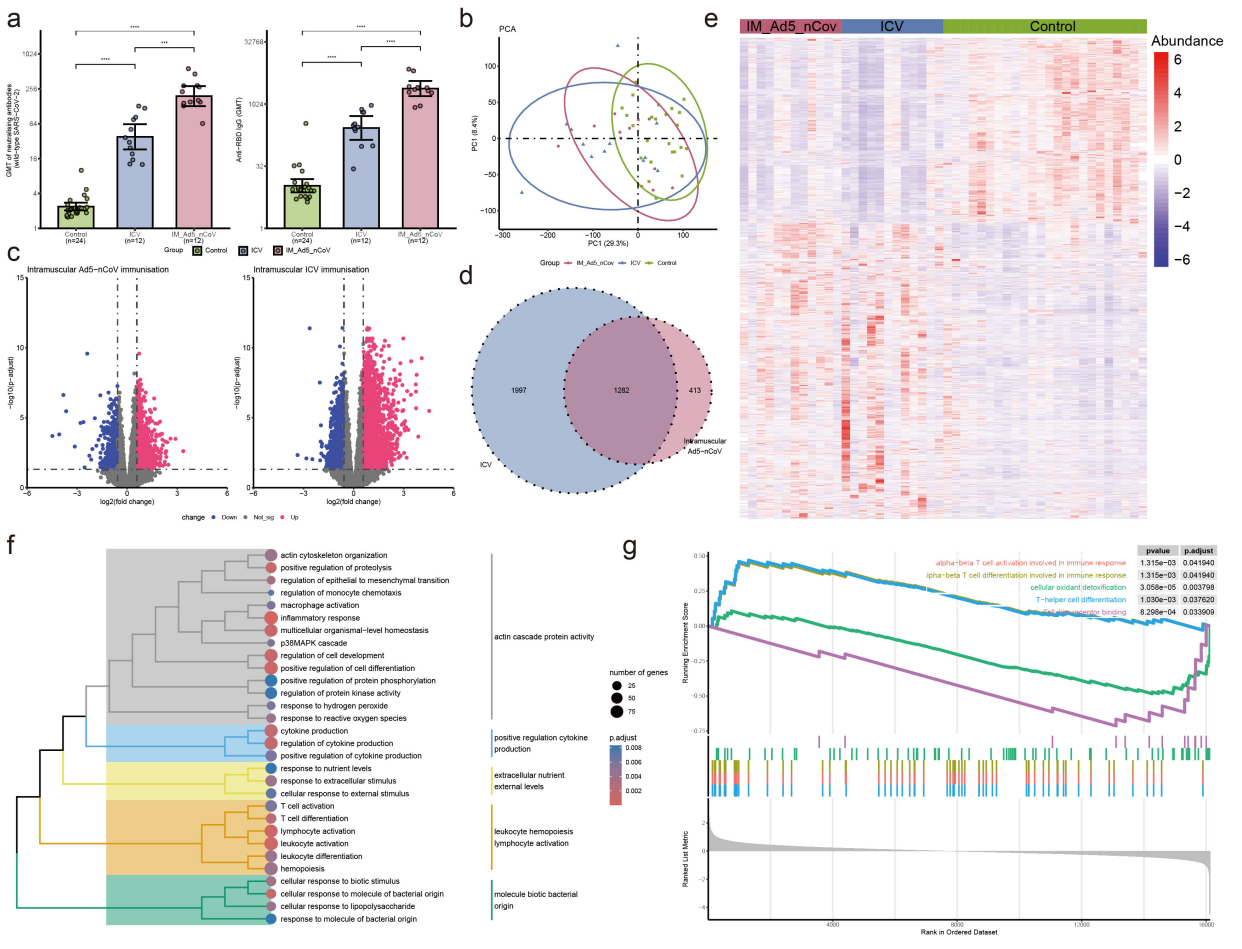
Neutralizing antibodies against wild-type SARS-CoV-2 and RBD-specific antibody and PBMC transcriptomic bioinformatics analysis of intramuscular Ad5-nCoV participants from cohort 1. **(A)** Neutralizing antibodies against wild-type SARS-CoV-2 and RBD-specific antibodies of intramuscular Ad5-nCov (IM-Ad5-nCov) and inactivated vaccine (ICV) participants. **(B)** Principal component analysis of PBMC transcripts in intramuscular Ad5-nCov participants. **(C)** Volcano plots showing differentially expressed genes (DEGs) before and after IM-Ad5-nCov or ICV vaccination. Blue, downregulated; red, upregulated. **(D)** Venn diagram showing DEGs before and after IM-Ad5-nCov and ICV vaccination. **(E)** Heatmap of DEGs before and after IM-Ad5-nCov and ICV vaccination. **(F)** Cluster analysis of Gene Ontology (GO) enrichment. Different colors in the tree diagram represent different enrichment modules. **(G)** Enrichment plots by Gene Set Enrichment Analysis (GSEA). ***p<0.001, ****p<0.0001.

**Figure 3:**
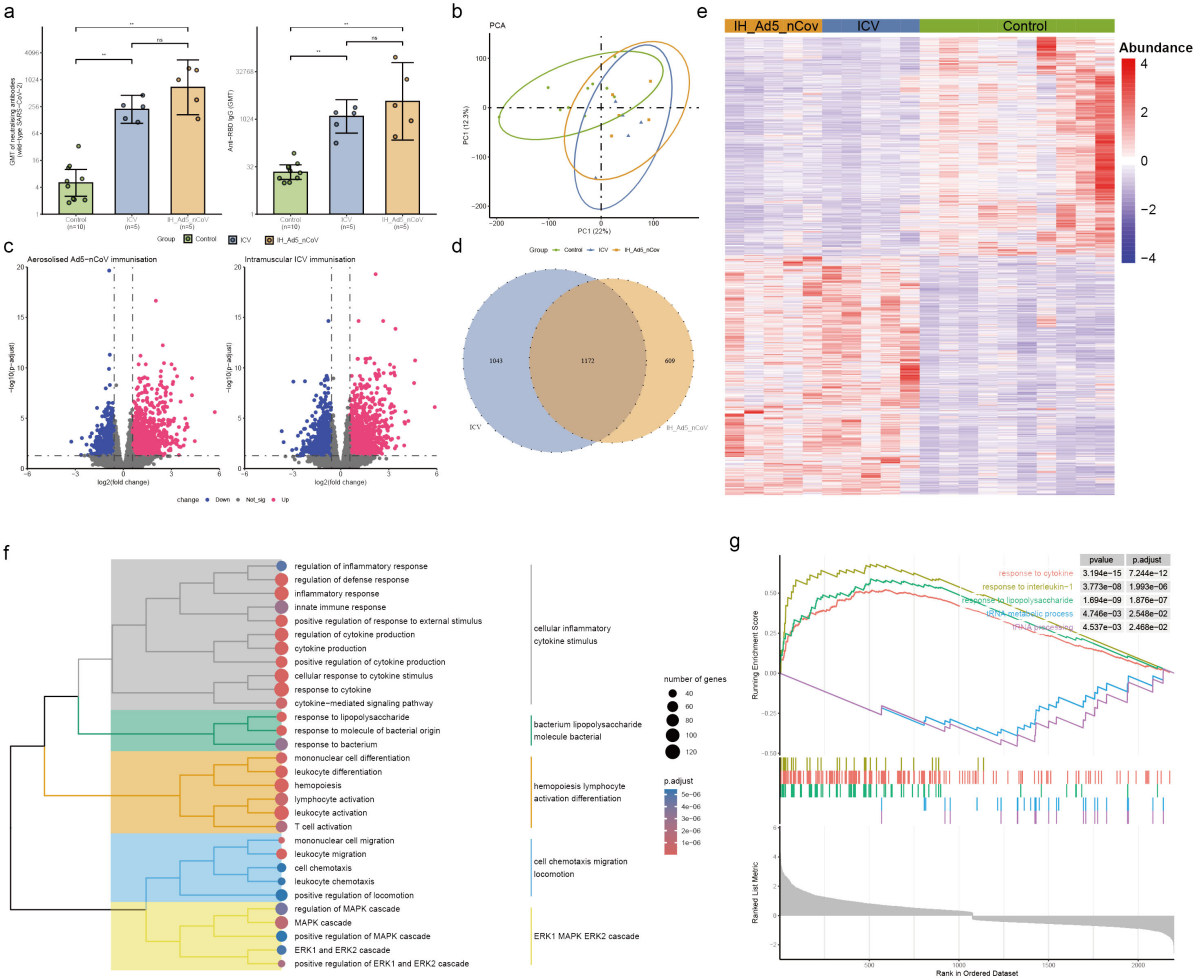
Neutralizing antibodies against wild-type SARS-CoV-2 and RBD-specific antibody and PBMC transcriptomic bioinformatics analysis of aerosolized inhaled Ad5-nCoV participants from cohort 2. **(A)** Neutralizing antibodies against wild-type SARS-CoV-2 and RBD-specific antibodies of aerosolized inhaled Ad5-nCov (IH-Ad5-nCov) and inactivated vaccine (ICV) participants. **(B)** Principal component analysis of PBMC transcripts in aerosolized inhaled Ad5-nCov participants. **(C)** Volcano plots showing differentially expressed genes (DEGs) before and after IH-Ad5-nCov or ICV vaccination. Blue, downregulated; red, upregulated. **(D)** Venn diagram showing DEGs before and after IH-Ad5-nCov and ICV vaccination. **(E)** Heatmap of DEGs before and after IH-Ad5-nCov and ICV vaccination. **(F)** Cluster analysis of Gene Ontology (GO) enrichment. Different colors in the tree diagram represent different enrichment modules. **(G)** Enrichment plots by Gene Set Enrichment Analysis (GSEA). **p<0.01, ns represents not significant.

The neutralizing antibody titers against wild-type SARS-CoV-2 observed before the third dose of vaccination were low and comparable across these groups: Geometric Mean Titer (GMT) of 4.3 (95% CI 1.2-15.4) in the aerosol Ad5-nCoV group, 2.7 (95% CI 2.0-3.6) in the intramuscular Ad5-nCoV group, 2.1 (95% CI 1.9, 2.4) in the ICV_1_ group and 5.5 (95% CI 2.9, 10.5) in the ICV_2_ group (**Table 2**). After 14 days of heterologous boosting, in cohort 1, the neutralizing antibody response observed in the intramuscular Ad5-nCoV group (191.8 [95% CI 129.0, 285.1]) was significantly higher than that in the ICV_1_ group (38.1 [95% CI 23.1, 62.8], p<0.0001; **Table 2**, **Figure 2A**); and in cohort 2, the aerosolized Ad5-nCoV group (738.4 [95% CI 250.9-2173.0]) a significantly higher neutralizing antibody response was also observed in the ICV_2_ group (244.0 [95% CI 135.0, 441.2], p=0.0434; **Table 2**, **Figure 3A**). The post-vaccination RBD-specific antibodies levels against wild-type SARS-CoV-2 were significantly higher in both the heterologous booster aerosolized Ad5-nCoV group (GMT of 4209.0 [95% CI 486.9-36384.0]) and the intramuscular Ad5-nCoV group (GMT of 2430.0 [95% CI 1595.0-3702.0]) compared to the ICV group (GMT of 270.0 [95% CI 138.8, 525.3] in the ICV_1_ group and GMT of 1403.0 [95% CI 534.7, 3681.0] in the ICV_2_ group) at day 14 (**Table 2**). The administration of the third dose of aerosol Ad5-nCoV resulted in a significant cellular response, as evidenced by IFN-γ and IL-13 ELISpot measurements at day 14 (**Table 2**). Participants in the aerosol Ad5-nCoV group exhibited a median IFN-γ+ spot count of 36.5 (IQR 15.3-58.8) and a median IL-13+ spot count of 22.5 (IQR 13.8-34.5) per 10^6^ PBMCs. In contrast, the intramuscular Ad5-nCoV and CoronaVac groups showed lower immunoreactivities with the third dose of vaccination.

**Table 2:** Neutralizing antibodies, RBD-specific IgG antibodies, and spike-specific T-cell cytokine responses before and after boosting.

	Cohort 1: NCT04892459			Cohort 2: NCT05043259		
	Ad5-nCoV-IM (n=12)	ICV_1_ (n=12)	P-value	Ad5-nCoV-IH (n=6)	ICV_2_ (n=6)	P-value
Neutralizing antibodies to wild type SARS-CoV-2						
Day 0						
GMT	2.7 (2.0, 3.6)	2.1 (1.9, 2.4)	0.1271	4.3 (1.2, 15.4)	5.5 (2.9, 10.5)	0.6624
Day 14						
GMT	191.8 (129.0, 285.1)	38.1 (23.1, 62.8)	<0.0001	738.4 (250.9, 2173.0)	244.0 (135.0, 441.2)	0.0434
Seroconversion rate (%)	100.0 (75.8, 100.0)	100.0 (75.8, 100.0)	1.0000	100.0 (61.0, 100.0)	100.0 (61.0, 100.0)	1.0000
GMFI	71.8 (52.4, 98.5)	18.0 (10.6, 30.4)	0.0001	172.5 (19.8, 1502.0)	44.4 (17.2, 114.7)	0.1704
Anti-RBD IgG antibody levels						
Day 0						
GMT	12.1 (6.1, 24.2)	9.5 (6.4, 14.2)	0.5136	30.0 (14.5, 62.2)	17.3 (9.2, 32.6)	0.1739
Day 14						
GMT	2430.0 (1595.0, 3702.0)	270.0 (138.8, 525.3)	<0.0001	4209.0 (486.9, 36384.0)	1403.0 (534.7, 3681.0)	0.2596
Seroconversion rate (%)	100.0 (75.8, 100.0)	100.0 (75.8, 100.0)	1.0000	100.0 (61.0, 100.0)	100.0 (61.0, 100.0)	1.0000
GMFI	200.4 (107.2, 374.4)	28.3 (17.1, 47.0)	<0.0001	140.3 (16.2, 1213.0)	81.0 (22.9, 286.4)	0.5846
IFN-r						
Day 0						
Mean ± SD	21.0 ± 12.7	9.3 ± 11.4	0.3135	2.5 ± 4.7	1.5 ± 1.0	0.6206
Median (IQR)	21.0 (12.0, 30.0)	5.0 (1.5, 21.3)		1.0 (0, 3.8)	1.5 (0.8, 2.3)	
Day 14						
Mean ± SD	16.5 ± 9.2	6.3 ± 4.4	0.1191	39.8 ± 28.7	3.7 ± 2.9	0.0118
Median (IQR)	16.5 (10.0, 23.0)	6 (2.3, 10.5)		36.5 (15.3, 58.8)	3.0 (1.0, 6.5)	
IL-13						
Day 0						
Mean ± SD	17.0 ± 14.1	7.0 ± 3.5	0.2072	5.3 ± 5.5	3.8 ± 2.6	0.5625
Median (IQR)	17.0 (7.0, 27.0)	7.0 (4.0,10.0)		4.5 (1.0, 7.8)	3.0 (1.8, 6.5)	
Day 14						
Mean ± SD	3.0 ± 1.4	7.5 ± 1.0	0.0097	24.7 ± 13.4	5.3 ± 4.5	0.0073
Median (IQR)	3.0. (0, 4.0)	8.0 (6.5, 8.0)		22.5 (13.8, 34.5)	4.0 (2.0, 8.0)	

GMT of neutralizing antibodies to wild-type SARS-CoV-2 and anti-RBD IgG. GMFI of neutralizing antibodies to wild-type SARS-CoV-2 and anti-RBD IgG. GMFI, geometric mean titer fold increase; GMT, geometric mean titer; ns, not significantly different; RBD, receptor-binding domain. Data are the Mean or median (IQR) of positive spot counts per 10^6^ PBMCs. *p-value is for comparisons among all groups.

### PBMC transcriptional features and pathways induced by intramuscular Ad5-nCoV enhanced vaccination

After intramuscular injection of Ad5-nCoV and inactivated vaccine, PCA analysis revealed significant changes in PBMC transcript levels in participants following booster immunization (**Figure 2B**). Analysis of gene transcript levels using the *DESeq2* package identified 942 up-regulated DEGs and 753 down-regulated DEGs after intramuscular injection of Ad5-nCoV, while 2131 up-regulated DEGs and 1148 down-regulated DEGs were observed with the inactivated vaccine (**Figure 2C**). Notably, 1282 DEGs were found to be common to intramuscular injections of Ad5-nCoV and the inactivated vaccine (**Figure 2D**), displaying a two-cluster distribution (**Figure 2E**). These findings suggest distinct gene expression patterns associated with the two vaccination strategies.

GO enrichment analysis of the DEGs following intramuscular injection of Ad5-nCoV revealed that these terms were grouped into five clusters: actin cascade protein activity, positive regulation cytokine production, extracellular nutrient external levels, leukocyte hemopoiesis lymphocyte activation, and molecule biotic bacterial origin (**Figure 2F**, **Supplementary Table 1**). Subsequent analysis using Gene Set Enrichment Analysis (GSEA) indicated that pathways related to alpha-beta T cell activation/differentiation in immune response, T-helper cell differentiation, and other T cell immune pathways were activated. Conversely, processes such as cellular oxidant detoxification, Toll-like receptor binding, and others were found to be inhibited (**Figure 2G**). This suggests a dynamic modulation of immune responses and cellular functions following intramuscular injection of the Ad5-nCoV vaccine. Furthermore, differential gene analysis of the inactivated vaccine group was mainly enriched in the response to differentiation metabolic and lymphocyte leukocyte activation adhesion (**Supplementary Figure 2**, **Supplementary Table 2**).

### PBMC transcriptional features and pathways induced by aerosolized inhaled Ad5-nCoV enhanced vaccination

To evaluate differences in transcript levels before and after aerosol-inhaled Ad5-nCoV or ICV vaccination, a principal component analysis (PCA) was conducted to reduce dimensionality and assess independence between groups (**Figure 3B**). The analysis revealed that both aerosol inhalation of Ad5-nCoV and ICV vaccination induced significant changes in PBMC transcript levels following the booster dose, compared to baseline levels. This suggests distinct alterations in gene expression profiles in response to the respective vaccination strategies. The differences in transcript levels between the aerosol inhalation Ad5-nCoV and ICV vaccination groups were found to be non-significant. Our analysis revealed that 1031 up-regulated DEGs and 750 down-regulated DEGs following aerosol inhalation of the Ad5-nCoV vaccine, while 1182 up-regulated DEGs and 1033 down-regulated DEGs were identified after intramuscular injection of the inactivated vaccine (**Figure 3C**). A Venn diagram illustrated 1172 (41.5%) common DEGs shared between aerosol inhaled Ad5-nCoV and intramuscular injection of the inactivated vaccine (**Figure 3D**), with these genes displaying a two-cluster distribution (**Figure 3E**).

GO enrichment analysis of the DEGs following aerosol inhaled Ad5-nCoV revealed that these terms were grouped into five clusters revealed that the terms were categorized into five main clusters: cellular inflammatory cytokine stimulus, bacterium lipopolysaccharide molecule bacterial, hemopoiesis lymphocyte activation differentiation, cell chemotaxis migration locomotion, and ERK1 MAPK ERK2 cascade (**Figure 3F**, **Supplementary Table 3**). Subsequent analysis using Gene Set Enrichment Analysis (GSEA) demonstrated a significant correlation with responses to cytokines, interleukin-1, and other immune pathways being activated after vaccination. Conversely, biological activities such as the tRNA metabolic process and tRNA processing were found to be inhibited (**Figure 3G**). This suggests a complex interplay of immune responses and cellular processes following vaccination. In addition, differential gene analysis of the inactivated vaccine group was mainly enriched in the immune activation differentiation bacterial and taxis mononuclear chemotaxis migration (**Supplementary Figure 3**, **Supplementary Table 4**).

Additionally, gene ontology (GO) analysis of the common transcriptional changes induced by aerosol inhalation/intramuscular injection of Ad5-nCoV and the inactivated vaccine CoronaVac as a third dose indicated that upregulated differentially expressed genes (DEGs) were enriched in immune-related pathways, specifically in protein kinase activity and inflammation regulation. Conversely, downregulated DEGs were enriched in the regulation of NF-kappaB transcription factor activity, melanosome assembly, and other functions (**Supplementary Figure 4**).

### Booster vaccine-induced expression patterns of PBMC genes associated with immune responses

**Figure 4:**
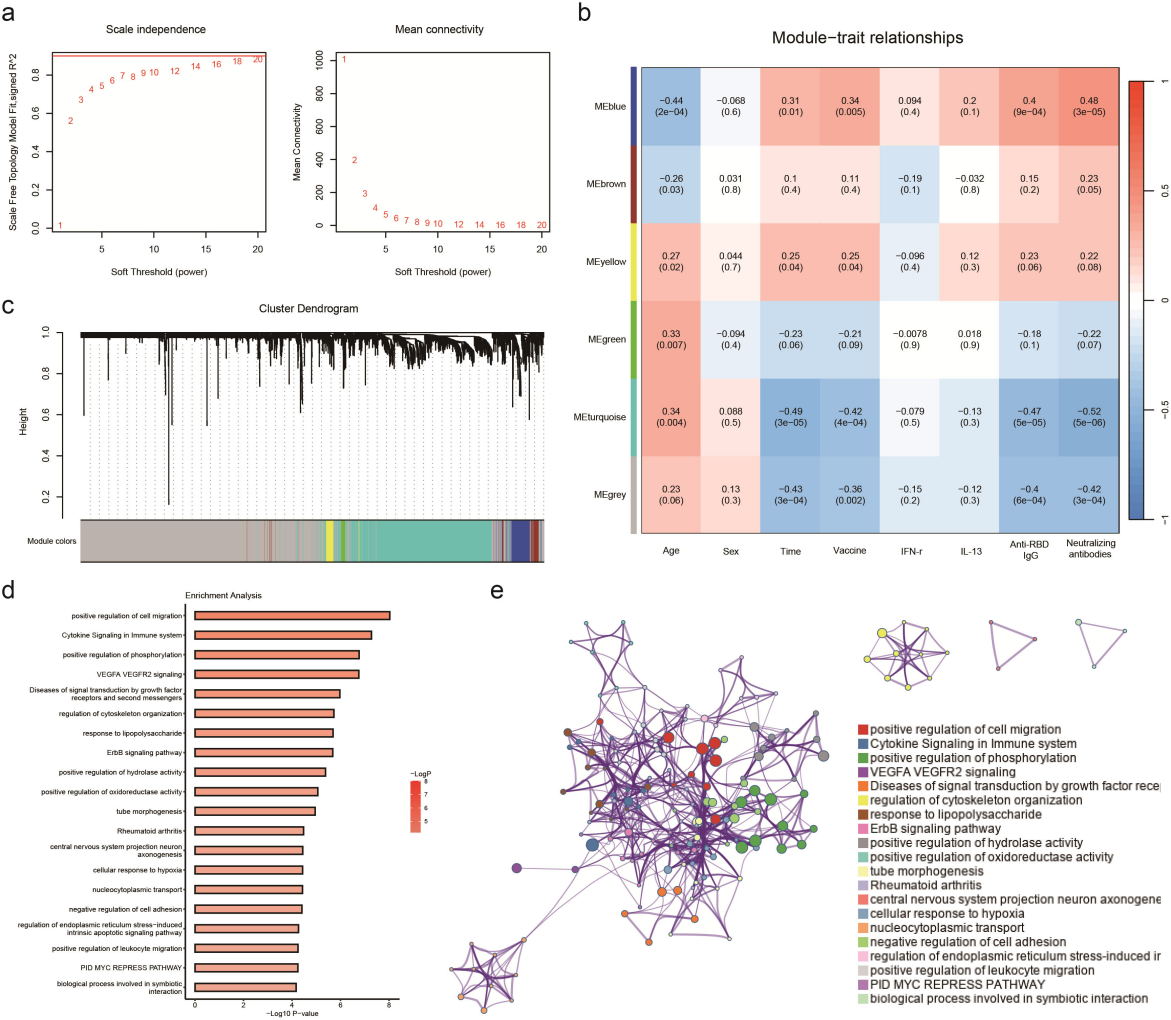
WGCNA analyses the transcriptome of participants receiving aerosol inhalation of Ad5-nCov (IH-Ad5-nCov), intramuscular injection of Ad5-nCov (IM-Ad5-nCov), and inactivated vaccine (ICV) to identify modules associated with clinical features and pathway enrichment. **(A)** Soft-thresholding calculation; Left: scale-free fit indices using various soft-thresholding powers; Right: mean connectivity using various soft-thresholding powers. **(B)** Heatmap of module-trait relationships. Each row corresponds to a module, and a column corresponds to a clinical trait. Each cell includes the corresponding correlation and p-value. **(C)** Cluster dendrograms of the coexpression network modules are ordered by hierarchical clustering of genes based on a 1-TOM matrix. Each module is colored differently. **(D)** Pathway enrichment analysis of modules (blue) significantly and positively associated with vaccination, wild-type SARS-CoV-2 neutralizing antibodies, and RBD-specific antibodies. **(E)** Relationships among these enrichment terms are displayed as a network (Metascape). Each term is represented by a circular node whose size is proportional to the number of input genes under the term and whose color represents its clustering identity (i.e., nodes of the same color belong to the same cluster). An edge connects terms with a similarity greater than 0.3 (the thickness of the edge represents the similarity score). The network was visualized with Cytoscape using a ‘force-directed’ layout, with the edges bundled for clarity. One term from each cluster was selected, and its term description was displayed as a label.

In this research, a soft threshold parameter of β = 16 was utilized to ensure the construction of scale-free networks (**Figure 4A**). Hierarchical clustering with a minimum module size of 30 identified a total of six modules, and the dendrogram was clustered based on similarity using 1-TOM (**Figure 4C**). Correlation analyses between vaccination characteristics and co-expression modules revealed a significant positive correlation between the blue module and vaccine type as well as neutralizing antibody titer (**Figure 4B**, **Supplementary Table 4**). The genes in the blue module were primarily associated with functions related to cell migration and immune cytokines, displaying a strong correlation within the network diagrams (**Figures 4D, E**). Conversely, the gray and turquoise modules exhibited a negative correlation with neutralizing antibodies, particularly in pathways such as adaptive immune response, cytoplasmic translation, and aerobic respiration (**Supplementary Tables 5, 6**).

## Discussion

Conventional neutralizing or epitope-based binding antibody profiles enable the direct assessment of the immunological effects of a vaccine, while comprehensive transcriptome data scanning and analysis provide insights into the underlying mechanisms of the host’s molecular immune responses to different vaccination series (18, 19). This study sheds light on the distinctive immune response elicited by heterologous boosting with aerosolized inhalation/intramuscular injection of Ad5-nCoV and homologous boosting with the inactivated vaccine CoronaVac as a third dose, considering both antibody characteristics and transcriptomics perspectives.

Both the humoral and cellular immunity data supported that the heterologous boosting Ad5-nCoV is more immunogenic than homologous CoronaVac immunization. The heterologous boosting of Ad5-nCoV by aerosol inhalation or intramuscular injection induced significantly higher titers of neutralizing antibodies against wild-type SARS-CoV-2 in individuals who were primed with two doses of the inactivated COVID-19 vaccine before than the homologous booster regimen of CoronaVac did. Moreover, significant enhancement of T-cell responses was noted when boosting with inhaled aerosolized Ad5-nCoV versus the inactivated vaccine CoronaVac, potentially contributing to long-lasting protection against SARS-CoV-2 (20, 21). These findings suggest that the heterologous boosting strategy with Ad5-nCoV is more immunogenic than the homologous CoronaVac immunization.

Transcriptome analysis of peripheral blood mononuclear cells (PBMCs) following the third booster dose revealed that the heterologous boosting of Ad5-nCoV by aerosolized inhalation or intramuscular injection induced fewer differentially expressed genes (DEGs) compared to the inactivated vaccine did. The co-upregulated DEGs in both groups were associated with functional pathways such as protein kinase activity and regulation of inflammatory responses, suggesting an activation of the immune system. Specifically, the pathways triggered by aerosol Ad5-nCoV predominantly involved immune-related processes, including cytokine generation, defense response, inflammatory response, and cytokine-mediated signaling pathways. On the other hand, intramuscular injection of Ad5-nCoV enriched immune-related pathways like cytokine production, T-cell activation and differentiation, leukocyte activation, and also induced pathways related to cell development regulation and positive regulation of protein hydrolysis. In previous single-cell studies, it was also demonstrated that vaccination with Ad5-nCoV activated not only immune-related pathways but also naïve and memory CD4+ T cells (22, 23). Memory CD4 T cells are required for long-lived immunity and are induced by vaccination strategies, including those against influenza (24). In comparison, the pathways enriched by the inactivated vaccines exhibited greater diversity, encompassing immune-related pathways such as leukocyte activation, cytokine response, immune response regulation, as well as positive regulation of cell differentiation, cell movement and migration, and hematopoietic function.

By comparing the performance of the above vaccines on the transcriptome, it was observed that the Ad5-nCoV vaccine more efficiently induced alterations in immune-related pathways in the nebulized inhalation modality than in the intramuscular injection modality, leading to the generation of higher levels of neutralizing antibodies. This finding further validates the superiority of the nebulized inhalation modality in eliciting vaccine-induced immune responses (4, 5). Similarly, in the intramuscular injection modality, the Ad5-nCoV vaccine demonstrated a greater ability to stimulate changes in immune-related pathways and produce elevated levels of neutralizing antibodies compared to the inactivated vaccine. This disparity may be linked to the specific antigenic components present in the vaccine formulations. Overall, diverse vaccination modes and types can yield varying effects on the body’s immune response, underscoring the significance of selecting appropriate vaccine types and administration methods in the context of vaccine development and implementation.

Weighted Gene Co-expression Network Analysis (WGCNA) is a powerful tool for identifying key molecular and cellular properties of protective immunity that are closely associated with sample parameters. In this study, the analysis revealed that the blue module was strongly positively correlated with levels of neutralizing antibodies and S-protein IgG antibodies while showing significant negative correlations with the turquoise and grey modules following the vaccine boosting. Genes in the blue module were found to be involved in innate immune regulatory functions, such as cytokine signaling in the immune system and positive regulation of leukocyte migration. On the other hand, genes in the turquoise and grey modules were associated with energy metabolism, carbohydrate synthesis, and other physiological functions. Previous studies on influenza, rubella, and yellow fever vaccines have also highlighted significant innate immune responses in the transcriptional regulation of vaccines, suggesting that the activation of the innate immune response may play a crucial role in the protective effects of vaccines. This underscores the importance of understanding the molecular mechanisms underlying immune responses to vaccines to enhance their efficacy and durability (12, 18, 25–27).

This study provides a comprehensive understanding of vaccine-induced immune responses through a systems biology approach (9, 11, 26, 27). However, there are some limitations of this study. Firstly, the results were assessed solely 14 days post-vaccination, neglecting the long-term characteristics and durability of the immune response. Second, vaccine-induced innate activation is typically transient and rapid, occurring within one to two days post-vaccination; this oversight regarding the initial vaccine-induced immune status necessitates greater emphasis in future studies. Furthermore, the study exclusively enrolled healthy individuals aged 18 to 59, omitting children and older adults, warranting further investigation into age-related immune response variances. Lastly, individuals who experienced COVID-19 infections post-heterologous and homologous booster immunizations were not part of the study, necessitating a more comprehensive exploration of immune response characteristics. These limitations need to be considered and addressed in future studies to more fully assess the effects of different vaccination modalities on immune responses and to provide a more reliable basis for developing more effective vaccination strategies.

## Conclusion

In this study, we systematically analyzed the characteristics of immune responses, including neutralizing antibodies, IgG antibodies, cytokines and high-throughput gene expression profiles, pre- and post-heterologous (inhaled Ad5-nCoV vaccine, intramuscular Ad5-nCoV vaccine) and homologous (inactivated vaccine) booster immunizations with the COVID-19 vaccine. The results indicated that heterologous booster immunization with the Ad5-nCoV vaccine produced a more favorable immune response than homologous vaccination. Nevertheless, caution is needed when assessing the protective effect of the vaccine and developing vaccination strategies.

## Data Availability

The data presented in the study are deposited in the Genome Sequence Archive in BIG Data Center (https://bigd.big.ac.cn/), Beijing Institute of Genomics (BIG), Chinese Academy of Sciences, accession number HRA008439.
