# Supplementary material for: Immune responses and transcription landscape of adults with the third dose of homologous and heterologous booster vaccines of COVID-19
Source: Front Immunol. 2024 Sep 12;15:1461419. doi: 10.3389/fimmu.2024.1461419 (PMC11424439; doi:10.3389/fimmu.2024.1461419)
Supplement: Supplementary file 1 [file DataSheet1.docx]

# Supplemental material


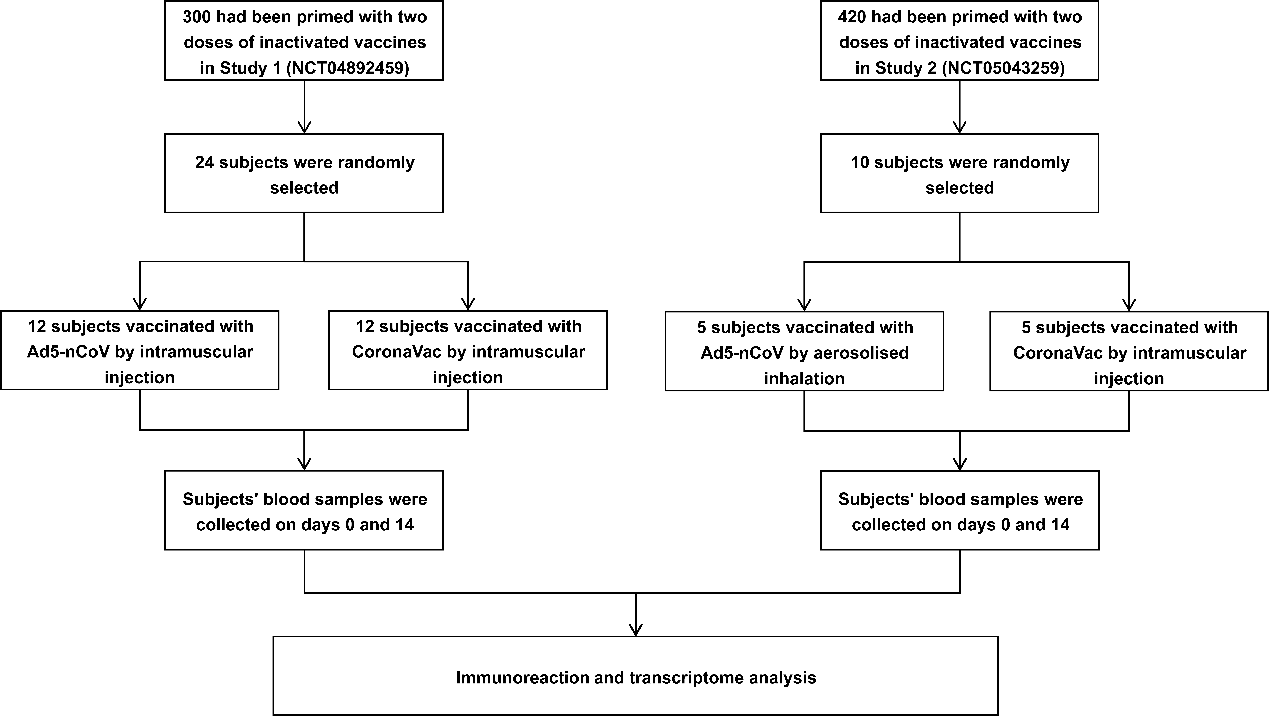


**Supplementary Fig. 1 Schematic of the sources of study participants.**


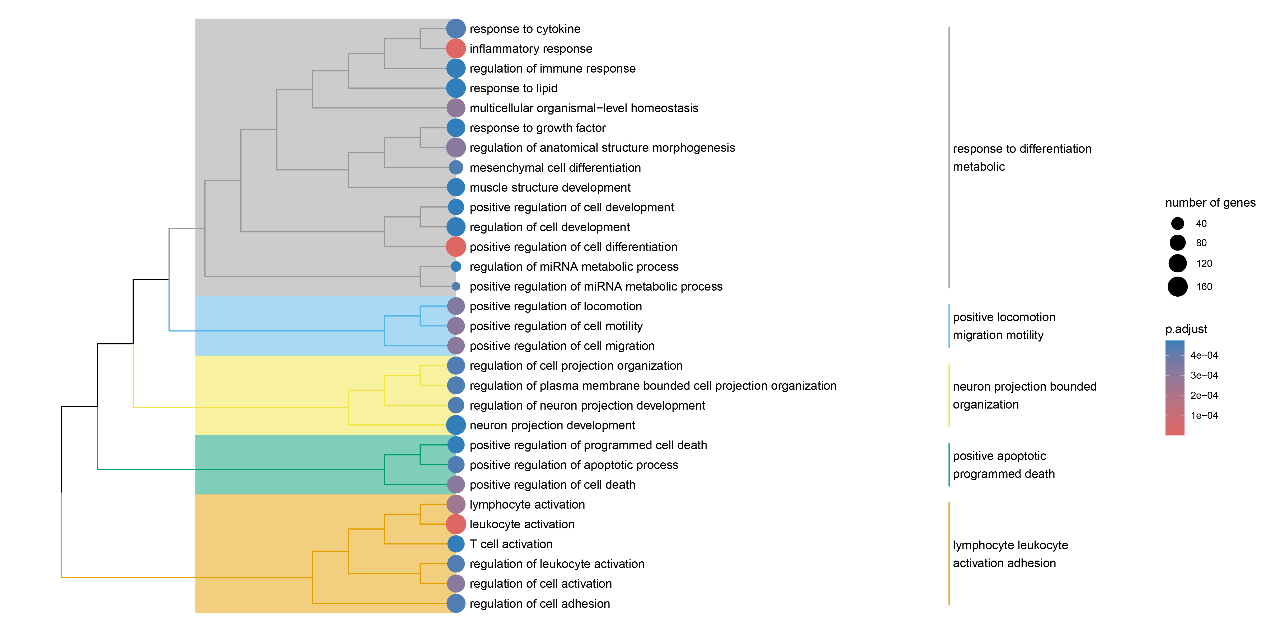


**Supplementary Fig. 2 Gene Ontology (GO) enrichment Clustering in participants with intramuscular injection of ICV from cohort 1.**

Different colors in the tree diagram represent different enrichment modules.


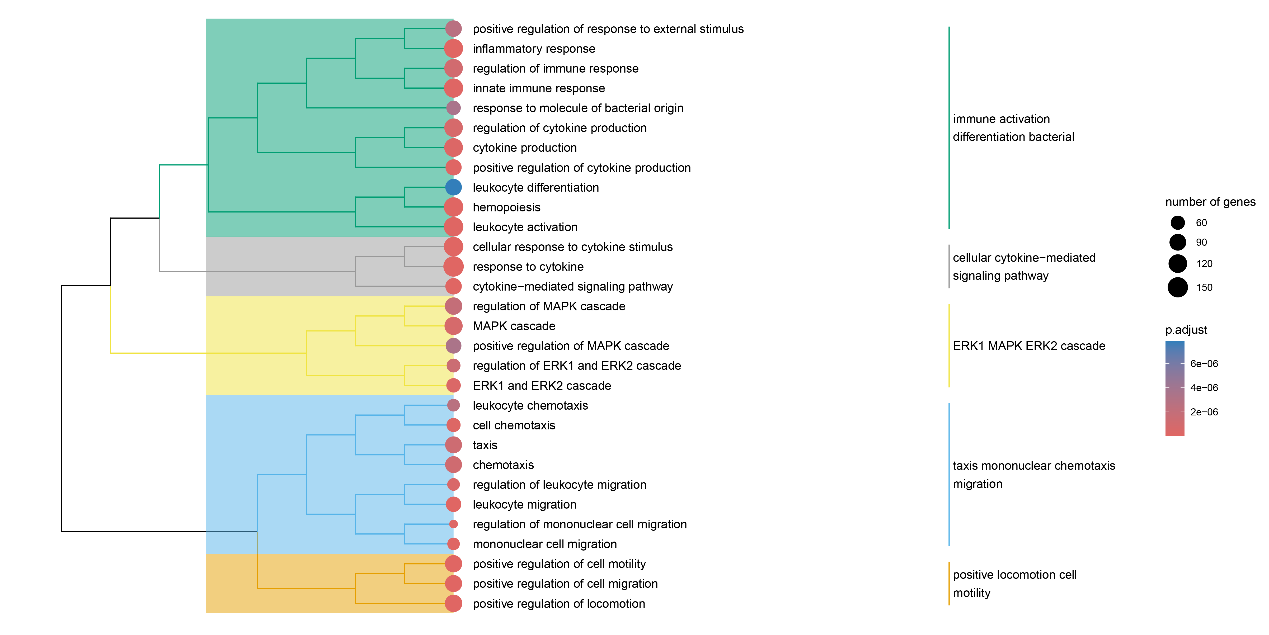


**Supplementary Fig. 3 Gene Ontology (GO) enrichment Clustering in participants with intramuscular injection of ICV from cohort 2.**

Different colors in the tree diagram represent different enrichment modules.


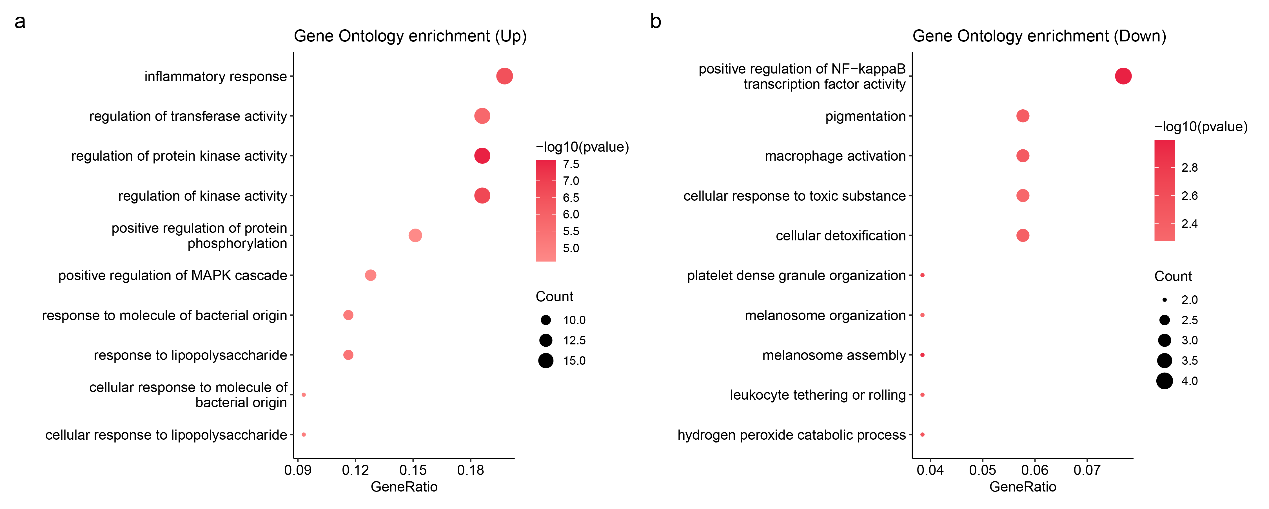


**Supplementary Fig. 4** **Gene Ontology (GO) enrichment of the common up or down-regulated transcriptional changes induced by the aerosolized inhalation/intramuscular injection of Ad5-nCoV and inactivated vaccine CoronaVac as a third dose.**

(a) Gene Ontology (GO) enrichment of the common up-regulated transcriptional changes; (b) Gene Ontology (GO) enrichment of the common down-regulated transcriptional changes.
